# mRNA Levels of Related *Abcb* Genes Change Opposite to Each Other upon Histone Deacetylase Inhibition in Drug-Resistant Rat Hepatoma Cells

**DOI:** 10.1371/journal.pone.0084915

**Published:** 2014-01-07

**Authors:** Ádám Sike, Enikő Nagy, Balázs Vedelek, Dávid Pusztai, Péter Szerémy, Anikó Venetianer, Imre M. Boros

**Affiliations:** 1 Department of Biochemistry and Molecular Biology, University of Szeged, Szeged, Hungary; 2 Institute of Biochemistry, Biological Research Center, Szeged, Hungary; 3 SOLVO Biotechnology, Szeged, Hungary; 4 Institute of Genetics, Biological Research Center, Szeged, Hungary; Indiana University School of Medicine, United States of America

## Abstract

The multidrug-resistant phenotype of tumor cells is acquired via an increased capability of drug efflux by ABC transporters and causes serious problems in cancer treatment. With the aim to uncover whether changes induced by epigenetic mechanisms in the expression level of drug transporter genes correlates with changes in the drug resistance phenotypes of resistant cells, we studied the expression of drug transporters in rat hepatoma cell lines. We found that of the three major rat ABC transporter genes *Abcb1a*, *Abcb1b* and *Abcc1* the activity of only *Abcb1b* increased significantly in colchicine-selected, drug-resistant cells. Increased transporter expression in drug-resistant cells results primarily from transcriptional activation. A change in histone modification at the regulatory regions of the chromosomally adjacent *Abcb1a* and *Abcb1b* genes differentially affects the levels of corresponding mRNAs. Transcriptional up- and down-regulation accompany an increase in acetylation levels of histone H3 lysine 9 at the promoter regions of *Abcb1b* and *Abcb1a,* respectively. Drug efflux activity, however, does not follow tightly the transcriptional activity of drug transporter genes in hepatoma cells. Our results point out the need for careful analysis of cause-and-effect relationships between changes in histone modification, drug transporter expression and drug resistance phenotypes.

## Introduction

Cancer chemotherapy is often impeded by development of the multidrug-resistant phenotype of tumors. Multidrug resistance (MDR) can arise by several mechanisms [1] that increase drug efflux by plasma membrane localized ABC transporters. MDR proteins gain energy by ATP hydrolysis to pump out a wide variety of molecules from the cells [2]. Different approaches have been attempted to hamper or eliminate drug resistance: among them is interfering with transporter function by small molecular inhibitors, peptides and antibodies, developing drugs that evade efflux [3], down-regulating *Abcb1* gene expression by blocking its transcription or translation via RNA interference [3].

Regulation of the expression of the major human drug transporter gene *Abcb1* is highly complex [4]. First, at the level of transcription initiation it involves several transcription factors and recruitment of histone acetyltransferase complexes. Second, alternative promoter usage [5] [6], promoter mutation [7] [8], tumor suppressors, oncogenes and stress conditions also influence the transcription of the *Abcb1* gene. Moreover, accumulating evidence indicates that histone deacetylase inhibitors, which are themselves promising anticancer agents, can also increase *Abcb1* expression, thus, contributing to the development of chemoresistance. On the other hand, transcriptional activation is not the only mechanism of ABCB1 overexpression. Gene amplification is often observed in highly drug-resistant cell lines [9] [10] and chromosomal translocation [11], and/or mRNA stabilization [12] can also contribute to the observed elevated ABCB1 protein levels.

In rodent genomes, two genes encoding homologues of the major human drug transporter ABCB1 are present. Both *Abcb1a* and *Abcb1b* are associated with the multidrug-resistant phenotype, but their differential overexpression was detected in multidrug-resistant cell lines [13], and in contrast to their high degree of similarities, mouse ABCB1 proteins differ in transport properties [14] [15] [16]. In primary sequence, ABCB1a is more closely related to the human homologue than ABCB1b. The regulatory region of *Abcb1a* also exhibits significant similarity with the human *Abcb1* gene [17] [18]. The transcriptional regulation of the two rat *Abcb1* genes has common and distinct characteristics. Differences can be observed in tissue specificity, as ABCB1a is the dominant form in the intestinal epithelium and at the blood-brain barrier, whereas ABCB1b is highly expressed in the pregnant uterus and in the ovaries [19]. Mice with *Abcb1a^−/−^*, *Abcb1b^−/−^* or double knockout genotypes are viable, fertile and show no physiological abnormalities revealing that under laboratory conditions these genes are not essential. However, increased penetration and reduced elimination of drugs were detected in many tissues of *Abcb1* mutants [20] [21].

The existence of two *Abcb1* homologues in rodents offers an opportunity for studying molecular causes of MDR phenotype development and in particular for the elucidation of the role of transcriptional control in this process. In this study, we analyzed the expression of the rat *Abcb1a* and *Abcb1b* genes in drug-sensitive and multidrug-resistant hepatoma cell lines. The mechanism underlying the overexpression of the *Abcb1* homologues was investigated with special interest in the role of histone acetylation. The drug-resistant clone 2 col500 (col500) and clone 2 col1000 (col1000) cell lines used in these experiments were selected from a dexamethasone-resistant hepatoma clone 2 (D12) by increasing the concentrations of colchicine. Previously, they have been shown to be resistant to structurally unrelated drugs and to overexpress *Abcb1* mRNAs [22] [23].

## Materials and Methods

### Cell Lines, Media, Culture Conditions

Rat hepatoma cell lines [22] [23] were maintained at 37°C in a humidified atmosphere of 5% CO_2_ and 95% air in complete medium (Ham’s F12 medium supplemented with 5% FCS, 1 mM L-glutamine, 0.01% streptomycin, 0.005% ampicillin). Multidrug-resistant col500 and col1000 cells were cultured in the presence of 500 or 1000 ng/ml of colchicine, respectively.

Cells used for DNA, RNA and protein extraction and to determine drug efflux activity were seeded in 6-well plates at 3×10^5^ cells/well in colchicine-free complete medium 3 days before the experiments.

When appropriate, the histone deacetylase inhibitor (HDACi) trichostatin-A (TSA) was added to the media in a 50 ng/ml final concentration for 6 h.

### Determination of the Multidrug Resistance Activity Factor (MAF)

The multidrug transporter activities were determined using flow cytometry and the MultiDrugQuant Assay kit (Solvo Biotechnology, Szeged, Hungary) [24]. The kit contains Calcein-AM as a probe substrate, Indomethacin as a selective ABCC1 inhibitor and Verapamil as an inhibitor for ABCB1 and ABCC1.

The assay is based on the following: Calcein-AM is a nonfluorescent membrane permeable molecule, which is a substrate of and removed from the cytoplasm by ABCB1 and ABCC1 transporters. In the cytoplasm, the acetoxi-methil ester bond of Calcein-AM is cleaved by nonspecific esterases resulting in the fluorescent, membrane impermeable molecule Calcein, which is not a substrate for transporters. Since these efflux transporters can remove the Calcein-AM prior to its cleavage, the transporter activity is detectable as the decrease in fluorescent intensity.

For MAF determination with TSA-treated cells, the HDACi was added to the culture at 50 ng/ml concentration for 6 h, then the media was changed and the cells further cultured for 4 h in order to permit protein expression. Following harvesting, cells were treated as indicated in the kit protocol.

Fluorescent intensity values were determined by flow cytometry (Partec CyFlow space) using a 488 nm excitation laser and a 512–542 nm detector, counting 10,000 cells/sample. Calcein multidrug resistance activity factors (MAF_C_) was calculated according to the following equation: **MAF_C_** = 100×(F_Max_–F_0_)/F_Max_, where F_Max_ is the maximum fluorescence of samples with Abcb1 and Abcc1 inhibitor (Verapamil); and F_0_ is samples with solvent control. Abcc1-related multidrug resistance activity factor: **MAF_Abcc1_** = 100×(F_Abcc1_–F_0_)/F_Max,_ where F_Abcc1_ is samples with Abcc1 inhibitor (Indomethacin). Abcb1-related multidrug resistance activity factor: MAF_Abcb1_ by subtracting MAF_Abcc1_ from MAF_C_: **MAF_Abcb1_** = MAF_C_–MAF_Abcc1_. The theoretical value of the multidrug resistance activity factors ranges between 0 and 100.

### RNA Extraction, Reverse Transcription and Quantitative Real-time PCR

Total RNA was isolated using RNeasy Plus Mini Kit (QIAGEN, Venlo, Netherlands) according to the manufacturer’s instructions. Two micrograms of total RNA was reverse transcribed with random hexamer primers using the TaqMan Reverse Transcription Kit (Applied Biosystems, California, USA) according to the manufacturer’s instructions. Quantitative real-time PCR was performed using Power SYBR Green technology with a 7500 Real Time PCR System (Applied Biosystems, California, USA). The following specific primers: rat *Abcb1a* (Abcb1a forward: 5′-ACCAGCGGTCAGTGTGCT-3′; Abcb1a reverse: 5′-CGGTTGTTTCCTACATTTGC-3′), rat *Abcb1b* (Abcb1b forward: 5′-GTTGGCATATTCGGGATGTT-3′; Abcb1b reverse: 5′-TCGCTGACGGTCTGTGTACT-3′), rat pre-*Abcb1a* (pre-Abcb1a forward: 5′-CCATGGAATTGCGCTCCCAC-3′; pre-Abcb1a reverse: 5′-GGACAGCCTCCACTACATAGACCA-3′) and rat pre-*Abcb1b* (pre-Abcb1b forward: 5′-TTCGGGATGGTGAGTTTGGGA-3′; pre-Abcb1b reverse: 5′-GGAAGCTCAGTACAGGGCAAAT-3′) were used to detect *Abcb1a, Abcb1b* mRNA and pre-mRNA sequences. Real-time PCR data were normalized to an 18S rRNA internal control and amplified using the 18S forward primer 5′-AAACGGCTACCACATCCAAG-3′ and 18S reverse primer 5′-CATCAACCTAGAACCCTCGC-3′.

### Genomic DNA Isolation and Quantitative Real-time PCR

Cells were harvested, washed, resuspended in lysis buffer (50 mM Tris-HCl pH 8.0, 10 mM EDTA, 2% SDS) and incubated at 65°C. Five minutes later, NaCl was added to the samples and the genomic DNA was precipitated with isopropanol. Real-time PCR was carried out using the following primers: Abcb1a 3. intron forward: 5′-CTTGCCCCACACTCTCATCT-3′, Abcb1a 3. intron reverse: 5′-TGAGACACTGGCTTTTCTGG-3′, Abcb1a end forward: 5′-GGGATCTGTAGGAAGAGGGG3′, Abcb1a end reverse: 5′-TCAAGTCTGCGTTCTGGATG-3′, Abcb1b 3. intron forward: 5′-ACTCCCTGCTCTGCATGTCT-3′, Abcb1b 3. intron reverse: 5′-AAAGGGTCTCTGGGTCAGGT-3′, Abcb1b end forward: 5′-GGTTATGGAGGGGGATCTGT-3′, Abcb1b end reverse: 5′-TCAAGTCTGCGTTCTGGATG-3′.

### Construction of *Abcb1* Regulatory Region Reporter Gene Plasmids

*Abcb1a* (+65 to −1520) and *Abcb1b* (+1 to −1448) regulatory regions were obtained by PCR amplification on a genomic DNA template prepared from D12 cells with primers *Abcb1a*: (5′-CCAAAAGGAAGGCAAGAACA-3′, 5′-CTTTCCGATTTCCTCCGTAA-3′) and *Abcb1b*: (5′-TTGCCTCTGTCCCTTCTGTT-3′, 5′-GAAGTCCGCCAAGATGTAGAA-3′). The amplified products were cloned into the luciferase reporter PGL3-basic vector (Promega, Wisconsin, USA) to generate pGL3-Abcb1a-Luc and pGL3-Abcb1b-Luc.

### Transfection and Luciferase Activity Measurement

pGL3-Abcb1a-Luc and pGL3-Abcb1b-Luc reporter plasmids were transfected into D12, col500 and col1000 cells using TurboFect reagent (Fermentas, Maryland, USA) according to the manufacturer’s instructions. After a 24 h incubation, the media was removed and the cells were washed and harvested. Cells were resuspended in 1x lysis buffer (Promega, Cell Culture lysis Reagent 5x) and incubated on ice for 30 min. Next, the samples were centrifugated at 13,000 rpm at 4°C for 5 min, and the supernatants were collected and used to determine protein concentrations by the Bradford method and to perform luciferase enzyme assays. Luciferase activity in the samples was determined using the Promega luciferase assay kit and the Orion L Microplate Luminometer (Berthold Detection System, Simplicity 4.2 software). To determine transfection efficiency, pEGFP-N3 was co-transfected, and the number of GFP-positive cells were determined by FACS (FACSCalibur) using an FL-1 filter.

### Comparison of RNA Stability

Cells were exposed to 20 µg/ml actinomycin D for 6, 12, 24 and 36 h at 37°C. After incubation, the media was removed and the cells were washed twice with ice-cold PBS. Total RNA isolation, reverse transcription and real-time PCR were carried out as described above.

### Western Blotting

For Western blots, total protein samples were run on 10% or 15% SDS-polyacrylamide gels and the proteins transferred to nitrocellulose membranes. The transfer efficiency was assessed by staining with 0.1% Ponceau solution. The membrane was washed, blocked with 5% non-fat dried milk in TBS-T buffer (50 mM Tris-HCl, 150 mM NaCl and 0.05% Tween 20, pH 7.4) and incubated with the primary antibody at 4°C. The primary antibodies anti-acetylated histone H4 (Serotec, AHP418) and anti-histone H3 (Abcam, ab1791) were diluted (1∶1000); anti-ATP-binding cassette, sub-family B, member 1A (Merck Millipore, AB10340), anti-Mdr1b (Merck Millipore, AB10337) and anti-α-tubulin (Sigma-Aldrich, T9026) were diluted (1∶5000) in TBS-T containing 1% non-fat dried milk. After extensive washing, the membranes were incubated with horseradish peroxidase-conjugated anti-rabbit Ig secondary antibody (DAKO, P0448) for 1.5 h at room temperature. Finally, the membranes were washed with TBS-T and the proteins were detected using the enhanced chemiluminescence detection system (ECL, Amersham Biosciences, Pittsburgh, USA).

### Chromatin Immunoprecipitation Assay

Approximately 5×10^6^ D12, col500 and col1000 cells were cultured on 10-cm plates in colchicine-free complete medium for 3 days. On the third day, the chromatin was cross-linked by adding formaldehyde to a 1% final concentration and shaking the culture for 7 min on ice. Cross-linking was terminated by adding glycine to a 0.125 M final concentration on ice. Cells were washed with PBS and harvested. Lysis buffer (1% SDS, 10 mM EDTA, 50 mM Tris-HCl pH 8.0, 500x PIC, 10 mM Na-butyrate) was added to the cells and incubated on ice for 30 min. Chromatin samples were fragmented by sonication with bioruptor (Diagenode; high throughput setting, 5 min in 30 s on/off intervals) in ice-cold water to an average length of 200 bp. Chromatin was precleared with a 50% slurry of previously blocked (1 µg/µl salmon sperm DNA, 3 µg/µl BSA) protein G-coated Sepharose beads (3 h, rotated at 4°C). Antibody [anti-histone H3 (Abcam, ab1791), anti-histone H3K9ac (Abcam, ab4441) and anti-histone H3K14ac (Upstate, 07–353), 2 µl of each] was added to the precleared chromatin samples in IP-buffer (150 mM NaCl, 20 mM Tris-HCl pH 8.0, 1% Triton X-100, 500x PIC, 10 mM Na-butyrate) and incubated with rotating at 4°C overnight. Next, 20 µl of a 50% slurry of blocked protein G-Sepharose was added to recover immune complexes (3 h at 4°C). Samples were centrifuged at 2,000 rpm for 2 min at 4°C. A 100 µl sample of no antibody control (NAC) supernatant was removed and used later as a total input control (TIC). Immunoprecipitates were washed three times with IP wash buffer 1 (0.1% SDS, 1% Triton X-100, 2 mM EDTA pH 8.0, 150 mM NaCl, 20 mM Tris-HCl pH 8.0), two times with IP wash buffer 2 (as above but 500 mM NaCl) and three times with IP wash buffer 3 (2 mM EDTA pH 8.0, 20 mM Tris-HCl pH 8.0, 10% glycerol). Pellets were resuspended in 100 µl TE buffer (10 mM Tris-HCl pH 8.0, 1 mM EDTA pH 8.0). Cross-linking was reversed by incubating the samples overnight at 65°C. Then, the proteins were digested by proteinase K (20 µg) at 37°C for 3 h in 0.5% SDS, the samples were extracted with phenol:chloroform:isoamyl-alcohol (25∶24:1) and the DNA was precipitated with ethanol. Pellets were resuspended in 50 µl Tris-HCl (pH 8.0) and analyzed by real-time PCR. The following primers were used: rat Abcb1a inic forward: 5′-GCCCAGGCACAGTCGAACA-3′; rat Abcb1a inic reverse: 5′-TCCTCCGTAAGGTCCGCCAA-3′; rat Abcb1a −1500 forward: 5′-CGCTCCTCATCAACCCATCCC-3′; rat Abcb1a −1500 reverse: 5′-ACCCAGGACTTCTTCGGCAC-3′; rat Abcb1b inic forward: 5′-TCCTTGCCCAATTCCACCCAC-3′; rat Abcb1b inic reverse: 5′-CAGCGGCCTCAGCCTCTTAC-3′; rat Abcb1b −1500 forward: 5′-TGTTTCTATGCCGCCCAAATCCA-3′; rat Abcb1b −1500 reverse: 5′-GCAAGGCCCCAACCCTTTCT-3′. The signal obtained with the no antibody control (NAC) was subtracted from all values before quantification.

### Statistical Analyses

Results are presented as mean ± SEM of the results of three or more independent experiments. Statistical analysis was done using SPSS for Windows version 15.0. Statistical significance was determined using the Mann Whitney U non-parametric test of significance with a p<0.05 considered statistically significant.

## Results

### Colchicine-selected, Drug-resistant Rat Hepatoma Cells Display Increased ABCB1 and Reduced ABCC1 Activity

**Figure 1:**
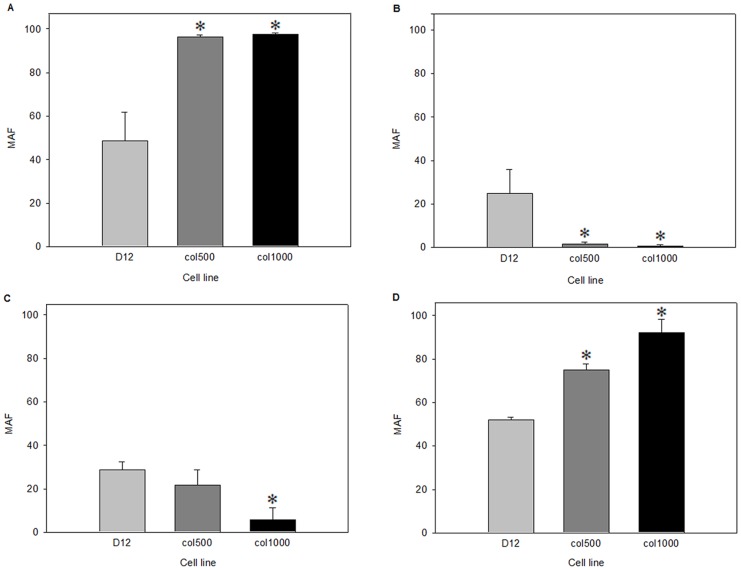
Activity of drug transporters in drug-sensitive and drug-resistant cell lines. MAF values reflecting ABCB1 (A), ABCC1 (B), ABCB1a (C) and ABCB1b (D) transporter activities were determined as described in the Materials and Methods and are shown as the percent of the total drug transporter activity of the indicated cell lines. (*p<0.05, Mann Whitney test).

The rat hepatoma cell lines, col500 and col1000, we used in these studies were selected by colchicine [22] [23], which is a substrate both for ABCB1 and ABCC1 [25]. Therefore, to reveal the contribution of particular transporters to drug resistance we first compared the drug removal capacity of drug-sensitive D12 and drug-resistant col500 and col1000 cells. For this, we determined the multidrug resistance activity factor (MAF) values [26] using the MultiDrugQuant Assay Kit by flow cytometric analysis. This technology permits the differentiation of multidrug resistance activity derived from ABCB1 or ABCC1 transporters. As expected, higher ABCB1 MAF values were detected in the drug-resistant cell lines compared to the drug-sensitive D12 cell line (Fig. 1A). Surprisingly, the ABCC1-derived MAF decreased significantly in the colchicine-selected cell lines (Fig. 1B).

Next, we selectively inhibited ABCB1b isoform activity by vinblastine [27] to distinguish between ABCB1a and ABCB1b activity. We found that the MAF value of ABCB1a decreased in the resistant col500 and col1000 cells as compared to the sensitive D12 (Fig. 1C). In contrast, ABCB1b activity was increased in both drug-resistant cell lines compared to the sensitive one (Fig. 1D). In summary, these data indicate that the ABCB1b transporter has the greatest contribution to the MDR phenotype of these drug-resistant cells.

### The mRNA Levels of both *Abcb1a* and *Abcb1b* are Increased in Drug-resistant Cells

**Figure 2:**
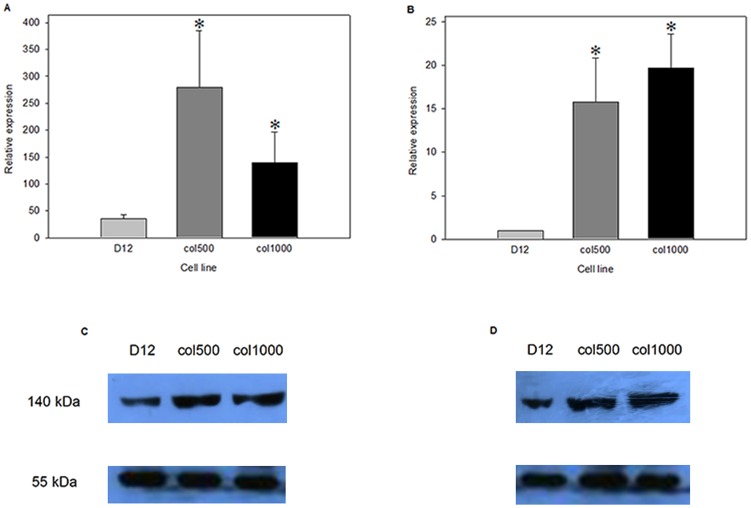
*Abcb1a* and *Abcb1b* mRNA and protein levels in drug-sensitive and resistant rat hepatoma cells. Relative mRNA levels of *Abcb1a* (A) and *Abcb1b* (B) in hepatoma cells were determined by quantitative real-time PCR. RNA samples were isolated from drug-sensitive parental (D12) and drug-resistant (col500 and col1000) cells, which were cultured for 3 days in the absence of colchicine. (*p<0.05, Mann Whitney test) The protein levels of ABCB1a (C) and ABCB1b (D) were determined by Western blot. Protein samples obtained from drug-sensitive and drug-resistant cells were separated on 10% SDS-polyacrylamide gels and transferred to immobilon membranes. Western blots were developed using ABCB1a and ABCB1b pan-specific primary antibodies. The loading control was developed with anti-tubulin Ab.

Since high drug efflux activity may result by several mechanisms affecting *Abcb1* gene expression and/or ABCB1 activity, we next determined the mRNA levels of *Abcb1a* and *Abcb1b* by quantitative RT-PCR. We found that in the parental D12 cell line *Abcb1a* and *Abcb1b* mRNA levels differed significantly with the level of *Abcb1a*-specific mRNA nearly thirty-fold higher than that of *Abcb1b* (Figs. 2A, B). In drug-resistant col500 and col1000 cell lines both genes were upregulated. Curiously, while the mRNA level of *Abcb1a* was higher than that of *Abcb1b* both in the parental and the resistant cell lines, the increase fold of *Abcb1b* expression in drug-resistant versus parental cells was considerably higher than that of *Abcb1a* (Figs. 2A, B).

### The Protein Levels of both ABCB1a and ABCB1b are Increased in Drug-resistant Cells

Western blotting analysis confirmed that ABCB1b is overexpressed in the resistant cell lines compared to the sensitive one (Fig. 2D), while the expression of ABCB1a differs modestly between sensitive and resistant cells (Fig. 2C). This observation is in accord with the results of mRNA level determination. In summary, the increase of *Abcb1b* expression in drug-resistant versus parental cells is considerably higher than that of *Abcb1a,* both in mRNA and in protein levels.

### Elevated *Abcb1* RNA Expression is not the Result of *Abcb1* Gene Amplification

**Figure 3:**
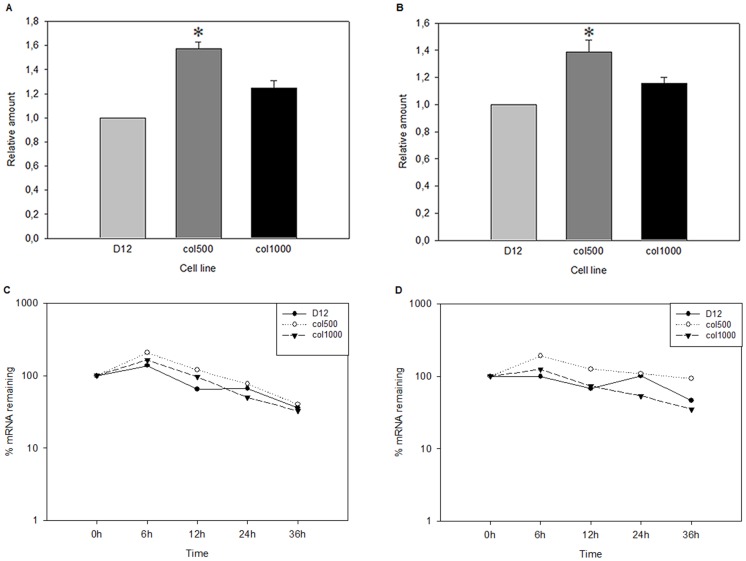
*Abcb1a* and *Abcb1b* gene copy numbers are unchanged, while the stability of *Abcb1a-* and *Abcb1b*-specific mRNAs differs slightly in drug-resistant cells. Gene copy numbers of *Abcb1a* (A) and *Abcb1b* (B) in a drug-sensitive parental cell line (D12) and drug-resistant cells (col500, col1000) were determined by PCR using gene-specific primers (see Materials and Methods). Genomic DNA was isolated from cells grown for 3 days in the absence of colchicine. (*p<0.05, Mann Whitney test). *Abcb1a* and *Abcb1b* mRNA stability was compared by quantitative real-time PCR on cDNA templates prepared from RNA samples, which were obtained from cells treated with 20 µg/ml actinomicyn D for 6, 12, 24 and 36 h, as indicated. The specific mRNA levels were compared to the levels of an 18S rRNA internal control. The change of mRNA levels of *Abcb1a* and *Abcb1b* over a 36-h period compared to the amount detected at zero time are shown in panels C and D, respectively.

One possible reason for *Abcb1* overexpression can be gene amplification in multidrug-resistant cells. In order to determine whether upregulated *Abcb1* mRNA levels in drug-resistant rat hepatoma cells were caused by the multiplication of the *MDR* locus, we compared the copy numbers of the *Abcb1* genes in drug-sensitive and drug-resistant cell lines. For this we performed quantitative PCR analysis on genomic DNA isolated from drug-sensitive and drug-resistant cells using *Abcb1a-* and *Abcb1b*-specific primers (Figs. 3A, B). We observed small fluctuations in the quantity of the *Abcb1* genomic regions, but this did not reach a two-fold difference and was within the sensitivity of the assay. Therefore, as the differences in mRNA levels were much higher, gene amplification alone was an unlikely cause of *Abcb1* overexpression in the studied rat hepatoma cell lines.

### mRNA Stabilization Contributes to *Abcb1b* Overexpression

Another possibility of increased *Abcb1* mRNA levels could be the stabilization of *Abcb1* mRNAs in the drug-resistant cell lines. To test this possibility, we compared the decay of *Abcb1* mRNAs by determining RNA levels 6, 12, 24 and 36 h after blocking transcription with actinomycin D treatment. We found that *Abcb1b* transcript levels in actinomycin D-treated col500 and col1000 cells were higher than in the parental D12 cell line at almost each time point tested (Fig. 3D). The observed differences in the decay of *Abcb1a* mRNA between drug-sensitive and resistant cell lines, on the other hand, were very modest (Fig. 3C). From these experiments, we concluded that an increase of mRNA half-life might contribute to elevated *Abcb1* mRNA levels and that this mechanism to a limited extent contributes to the differential expression of the two adjacent rat *Abcb1* genes.

### Transcriptional Activation is the Primary cause of Increased *Abcb1a* and *Abcb1b* Expression

**Figure 4:**
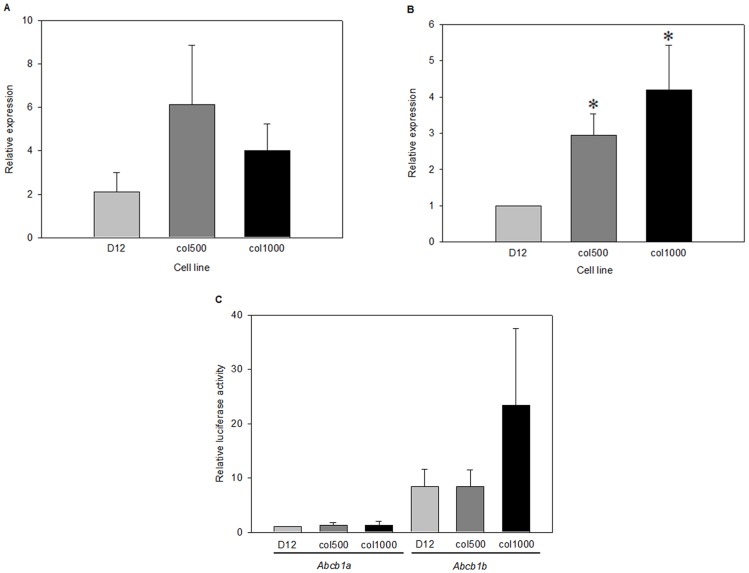
*Abcb1* pre-mRNA levels and promoter activities in drug-sensitive and resistant cell lines. The pre-mRNA levels of *Abcb1a* (A) and *Abcb1b* (B) were determined by quantitative PCR using specific primers hybridizing to intronic regions of the corresponding gene. RNA samples for cDNA preparation were obtained from cell cultured for 3 days in the absence of colchicine. The RNA levels were compared to the level of 18S rRNA internal control. (*p<0.05, Mann Whitney test). Luciferase activity determined in cell extracts prepared from D12, col500 and col1000 cells transiently transfected with the pGL3-Abcb1a-Luc or pGL3-Abcb1b-Luc reporter plasmids (C).

Transcription initiation control is one of the most frequent mechanisms of gene expression regulation, and *Abcb1* expression is known to be under complex transcriptional control. Therefore, after testing the possible role of gene amplification and mRNA stabilization we compared the transcription rate of *Abcb1a* and *Abcb1b* genes in drug-sensitive and drug-resistant cell lines. For this we used quantitative RT-PCR to measure the level of unprocessed *Abcb1a* and *Abcb1b* pre-mRNAs since unspliced transcripts have a short half-life and their quantity reflects the intensity of transcription. To detect the presence of pre-mRNAs, we used primers designated to hybridize to the 4^th^ exon and 4^th^ intron of *Abcb1a* and to the 3^rd^ exon and 3^rd^ intron of *Abcb1b*. As a result of the positions of the primers, the products of these PCR reactions could result only from the unspliced pre-mRNAs. By these experiments, we detected elevated pre-*Abcb1a* and pre-*Abcb1b* levels in col500 and col1000 cell lines compared to the parental D12 cells (Figs. 4A, B). Thus, these data, similar to those obtained by comparing mRNA levels, suggest that *Abcb1* genes are transcriptionally up-regulated in drug-resistant cells.

### *Abcb1* Promoter Fragments show Similar Activity in the Drug-sensitive and Drug-Resistant Cell Lines

In order to identify the regulatory elements participating in the transcriptional up-regulation of *Abcb1* genes in drug-resistant cells, we constructed plasmids in which the transcription start site (TSS) upstream regions of *Abcb1a* or *Abcb1b* were inserted in front of a reporter gene. For both genes, we inserted fragments carrying approximately 1,500-bp TSS-upstream fragments in front of luciferase coding sequence. The promoter-reporter fusion plasmid constructs were transiently transfected into drug-sensitive D12 and drug-resistant col500 and col1000 cells and luciferase reporter activity was determined. We did not detect significant differences in the promoter activity of *Abcb1a-* and *Abcb1b*-specific fragments in different cell lines (Fig. 4C). This might suggest that either the regions providing distinct activities to the two genes are located outside of the used promoter fragments, or that the strength of the promoters is influenced by the chromatin structure, which is not reproduced by the plasmid constructs used in transient expression. Interestingly, the promoter strength of *Abcb1* genes, as reflected by the detected reporter activity in transient expression assays, did not correlate with the *in vivo* mRNA levels of *Abcb1*-specific mRNAs either. The promoter of the *Abcb1b* gene drives a higher reporter gene expression, while the *Abcb1a* gene has a higher mRNA level *in vivo*. This observation might also indicate the contribution of epigenetic mechanisms to *Abcb1* gene expression.

### The Regulatory Regions of the *Abcb1* Genes Display Similar Levels of Acetylated Lysine 9 Histone H3 in Drug-sensitive and Drug-resistant Cell Lines

**Figure 5:**
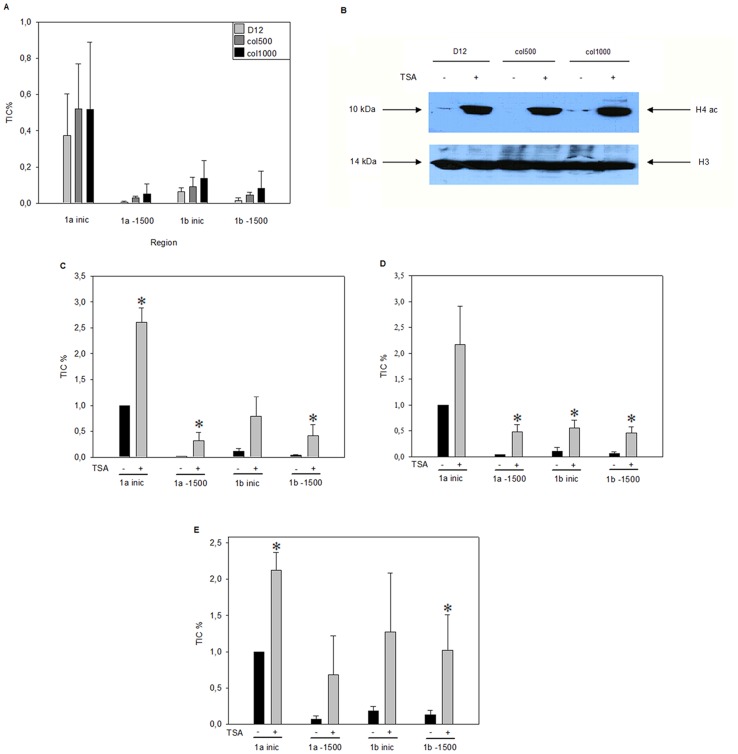
TSA treatment increases H3K9ac levels at the 5 ′ region of both rat *Abcb1* genes. (A) Detection of H3K9ac in drug-sensitive (D12) and resistant (col500, col1000) cells at the transcription initiation site (-inic) and 1500 nucleotides upstream of that (−1500) in the *Abcb1a* (a-) and *Abcb1b* (b-) regulatory regions by ChIP experiments. Primers and antibodies used are described in the Materials and Methods. (B) TSA treatment results in a comparable increase in acetylated histone H4 levels in both drug-sensitive and resistant cells. Protein samples obtained from TSA-treated (6 h, 50 ng/ml) and untreated, drug-sensitive and drug-resistant cells were separated on 15% SDS-polyacrylamide gels and transferred to immobilon membranes. Western blots were developed using acetylated histone H4 pan-specific primary antibody. The loading control was developed with anti-H3 Ab. (C–E) TSA treatment similarly affects H3K9ac levels in the *Abcb1a* and *Abcb1b* 5′ regions in drug-sensitive (D12; C) and drug-resistant (col500; D and col1000; E) cells. ChIP experiments were performed to detect H3K9ac levels as described in the Materials and Methods. (*p<0.05, Mann Whitney test).

Since histone modifications, and specifically acetylations, represent one of the most frequent chromatin marks contributing to transcriptional regulation, we wanted to determine whether *Abcb1* gene regulatory regions differed in this respect. We compared the presence of an acetylated histone form H3K9ac (lysine 9 acetylated histone H3), which is known to be frequently associated with transcriptionally activated regions. We examined the levels of H3K9ac at the transcriptional start sites and at around −1500 of the *Abcb1a* and *Abcb1b* genes by chromatin immunoprecipitation. Although we observed significant differences in H3K9ac levels between the two tested regions of the *Abcb1* genes, we detected no significant differences in H3K9ac levels in either region between drug-sensitive and drug-resistant cell lines, in sharp contrast to the fact that they displayed very different levels of *Abcb1* expression (Fig. 5A). By performing ChIP experiments on D12 drug-sensitive and col1000 highly drug-resistant cells with anti-H3K14ac antibodies, we obtained data qualitatively identical to the above, such as no differences were detectable in the levels of K14 acetylated histone H3 at the *Abcb1* 5′ regions in drug-resistant and sensitive cell lines (data not shown).

### The Two *Abcb1* Genes Respond Differently to Histone Deacetylase Inhibitor Treatment

**Figure 6:**
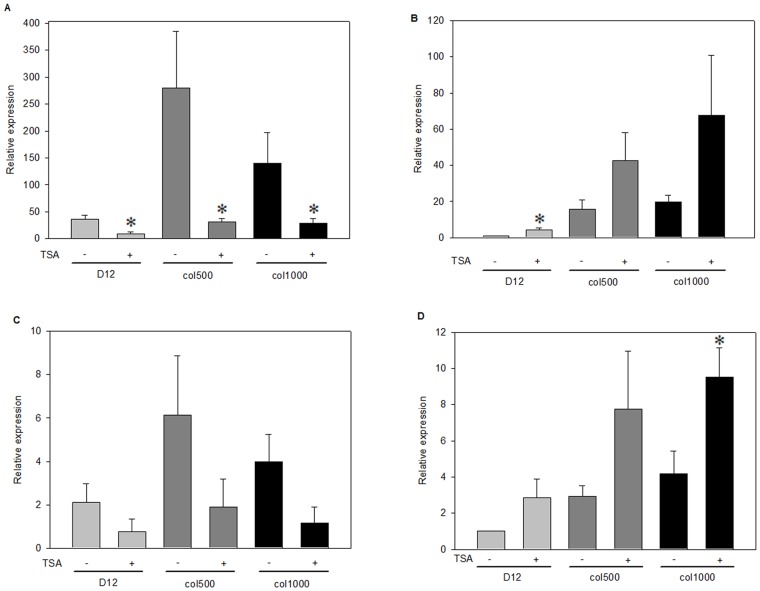
*Abcb1a and Abcb1b* mRNA and pre-mRNA levels are differentially altered by TSA treatment. *Abcb1a* (A and C) and *Abcb1b* (B and D) specific mRNA (A and B) and pre-mRNA (C and D) levels were compared in TSA-untreated and TSA-treated, drug-sensitive (D12) and drug-resistant (col500 and col1000) cells by quantitative PCR as described above. (*p<0.05, Mann Whitney test).

Finding no differences in H3K9ac and H3K14ac levels at *Abcb1* genes in drug-sensitive and resistant cells made us wonder whether histone acetylation affected ABCB1 expression at all. In order to test the regulatory potential of histone acetylation on the expression of *Abcb1* genes, we compared *Abcb1* expression in the absence and presence of histone deacetylase (HDAC) inhibitor trichostatin A (TSA). The efficacy of histone deacetylase inhibition was verified by Western blots showing that acetylated histone H4 levels were higher in both drug-sensitive and drug-resistant cell lines (Fig. 5B) after TSA administration. Results of chromatin immunoprecipitation experiments demonstrated that TSA treatment resulted in elevated H3K9ac (and also K14) levels at the *Abcb1a* and *Abcb1b* genes as well, with both tested 5′ regions. Significantly, the elevation in acetylated H3K9 levels was clearly detectable in both the drug-sensitive and drug-resistant cell lines (Figs. 5C, D, E). However, when we examined the effect of TSA on the expression of the *Abcb1* genes, we found that the two genes responded differently to the HDAC inhibitor: the mRNA level of *Abcb1a* decreased, while that of *Abcb1b* increased (Figs. 6A, B). We observed a different behavior of the two MDR genes in response to HDAC inhibition in the parental and the drug-resistant cell lines as well. A comparison of *Abcb1* pre-mRNA levels in TSA-treated versus untreated D12 parental and col500 and col1000 drug-resistant cells indicated similar changes in *Abcb1* expression upon TSA treatment, demonstrating that the histone deacetylase inhibitor affected the transcription of the two *Abcb1* genes differently (Figs. 6C, D).

### ABCB1 Activity Remains Unchanged Upon Histone Deacetylase Inhibitor Treatment

Finding that TSA treatment altered the expression levels of the *Abcb1* genes, we tested whether it also affected the drug-accumulating capacity of the cells. For this, the drug efflux capacity of TSA-treated and untreated D12, col500 and col1000 cells were compared by flow cytometric analysis using the MultiDrugQuant Assay Kit. TSA administration altered neither the ABCB1 nor ABCC1 activity of the drug-sensitive or drug-resistant cells (data not shown). In short, although inhibition of histone deacetylation by TSA-treatment resulted in changes in *Abcb1* expression, it did not significantly influence the drug efflux activity of rat hepatoma cells.

## Discussion

We report here the expression of *Abcb1a* and *Abcb1b* ABC transporter-coding genes in multidrug-resistant rat hepatoma cell lines. In particular, we wanted to uncover whether epigenetic mechanisms contribute to the overexpression of the major rat drug transporters ABCB1a and ABCB1b in drug-resistant cells. Furthermore, we wanted to determine whether changes in the expression level of these genes correlates with changes of drug resistance phenotypes. The multidrug-resistant cell lines we used in these studies were selected previously from a rat hepatoma clone (D12) using increasing concentrations of colchicine [22] [23]. Earlier data showed that MDR proteins (P-gp) were overexpressed in these multidrug-resistant cell lines, but the two *Abcb1* homologues were not analyzed separately [22] [23].

**Figure 7:**
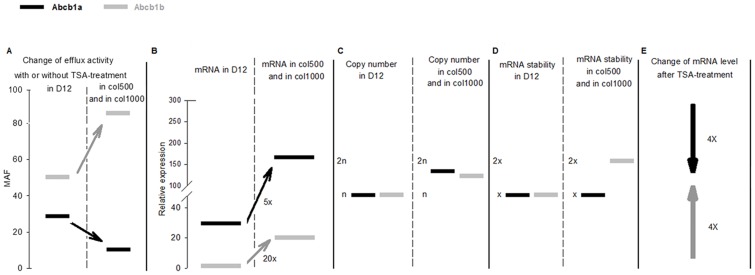
Summary of the drug efflux and drug-transporter expression changes observed in drug-selected rat hepatoma cells. (A) The major contributor to the drug efflux increase in drug-resistant rat hepatoma cell lines is their increased ABCB1b activity. (B) The mRNA levels of the *Abcb1a* and *Abcb1b* transporters differ significantly in the parental D12 cells, and are up-regulated differently in drug-selected cell lines. (C) Changes in copy numbers of neither *Abcb1a* nor *Abcb1b* are responsible for their elevated expression. (D) *Abcb1b* mRNA stability is slightly higher in the drug-resistant col500 and col1000 cells compared to D12. (E) HDAC inhibition affects *Abcb1* gene expression differently: upon TSA- treatment *Abcb1a* mRNA levels decrease, while *Abcb1b* mRNA levels increase both in drug-sensitive and drug-resistant cells.

We found that the drug-efflux ability of the resistant cells (col500 and col1000) results primarily from the activity of the ABCB1b transporter (Fig. 7A). In contrast, both *Abcb1a* and *Abcb1b* transcripts were overexpressed in the drug-resistant cell lines (Fig. 7B). The parental D12 cells also expressed *Abcb1a* and *Abcb1b* in substantial amounts, which can be explained by their hepatoma origin and dexamethasone resistance because hepatocarcinogenesis and dexamethasone treatment have been associated with the activation of *Abcb1* genes in rodents [28] [29] [30] [31] [32]. In accordance with its greater contribution to transporter activity, the increase both in the mRNA and in protein level of *Abcb1b* was higher in the multidrug-resistant cells than that of *Abcb1a*; although the latter had a higher absolute mRNA level both in the parental and the colchicine-selected cells (Fig. 7B).

Searching for the mechanisms underlying *Abcb1* overexpression, we compared the copy numbers of *Abcb1* genes and the stability of their mRNA products. Both amplification of MDR genomic regions and mRNA stabilization was suggested as the primary mechanism responsible for the development of the multidrug phenotype [9] [10]. However, we could not detect increased copy numbers of either *Abcb1a* or *Abcb1b* genes (Fig. 7C) and detected only a small increase in *Abcb1b* mRNA stability (Fig. 7D). Therefore, changes in copy number and/or mRNA stabilization might contribute to the elevated expression of the *Abcb1b* gene in drug-resistant cells but cannot explain the detected 5–20-fold increase in mRNA levels. Rather, transcriptional activation seems to be the primary reason for up-regulation of the *Abcb1a* and *Abcb1b* genes in these multidrug-resistant cells as indicated directly by elevated pre-mRNA levels of *Abcb1a* and *Abcb1b*. Transient expression of reporter gene linked *Abcb1* promoter fragments did not modify expression in drug-resistant and sensitive cell lines. This is, however, not surprising, since in the plasmid constructs the promoter fragments are removed from their chromosomal environment, therefore, distant regulatory elements and epigenetic mechanisms acting through modification of chromatin organization can hardly influence transcription from these promoters. That an epigenetic mechanism could have key roles in *MDR* gene expression was shown by previous studies, which demonstrated that hypermethylation and a low level of histone acetylation ensured transcriptional repression in drug-sensitive cell lines [5] [33]. Since the studied rat *Abcb1* genes showed expression in the drug-sensitive parental cell line, we did not consider DNA methylation in this study. Instead, we examined the possible regulatory role of histone acetylation. Acetylation of H3K9 and H3K14 are histone modification forms associated most frequently with transcriptional activation. Genomewide studies revealed that H3K9ac and H3K14ac are enriched around transcriptional start sites, at promoters and other regulatory elements of actively transcribed genes [34] [35] [36] [37]. We compared the levels of H3K9ac and H3K14ac in drug-sensitive and drug-resistant cell lines at the TSS and upstream of that in *Abcb1a* and *Abcb1b* but found no significant differences in this respect between drug-sensitive and resistant cell lines. Nonetheless, both genes were up-regulated in the drug-resistant cells. Consequently, the up-regulated expression of *Abcb1* genes in drug-resistant cells is not correlated with increased H3K9/K14 acetylation.

Although H3K9/K14 acetylation was not associated with *Abcb1* up-regulation, inhibition of histone deacetylation affected *Abcb1* transcription markedly: the mRNA level of *Abcb1a* decreased, while the mRNA level of *Abcb1b* increased after TSA treatment. The changes of pre-mRNA levels suggest that these changes resulted from transcriptional down-regulation of *Abcb1a* and transcriptional up-regulation of *Abcb1b*. Histone deacetylase inhibitors are potential anticancer agents that are under clinical trials [38]. Therefore, understanding their effect on *Abcb1* transcription and, thus, in MDR development is an important issue. Several studies showed a cell type specific up-regulation of *Abcb1* expression by histone deacetylase inhibitors [39] [40] [41] [42] [43] [44]. Here, we compared the effect of TSA on the two rat *Abcb1* homologues in drug-sensitive and drug-resistant cell lines. As expected, the administration of TSA increased global histone H4 acetylation and resulted in elevated levels of H3K9ac/K14ac at the *Abcb1a* and *Abcb1b* genes, both in drug-sensitive and drug-resistant cells. Despite that and of their similar function, the transcription of the two genes was affected differently (Fig. 7E). Their different behavior emphasizes the diverse regulation of their transcription. As we detected the different response of the two genes in both drug-sensitive and drug-resistant cells, our results differ from those of El-Khoury et al. [42] who have found that *Abcb1* expression was up-regulated only in drug-sensitive (H69WT) but not in etoposide-resistant (H69VP) cells upon TSA treatment. This might indicate that transcription responses are cell-type specific both in drug-sensitive and drug-resistant cell lines.

Although TSA treatment altered the expression of the two *Abcb1* genes, it did not significantly affect the drug-efflux potential of the cells. One explanation for this could be that the opposite changes in expression compensate for each other. However, the involvement of other drug transporters potentially affected by TSA cannot be excluded either.

Our findings clearly indicate that elevations of the level of histone acetylation are not necessarily accompanied by increased *Abcb1* gene expression. The expression of the two rat *Abcb1* genes in the studied hepatoma cells does not change similarly with alterations of the histone acetylation levels at their regulatory regions. El-Khoury et al. also reported [42] that TSA treatment failed to increase the expression of *Abcb1* in the etoposide-resistant cell line (H69VP), although acetylation levels increased on the gene. Recently, Toth et al. have shown that the expression levels of MDR genes were mostly uncoupled from the histone acetylation state in a drug-resistant breast cancer cell line [45]. While this seems surprising, in light of the numerous reports that an increase in histone acetylation levels can cause up- and down-regulation of a comparable number of genes, it is not so unexpected. It is more curious that in the case of the two rat *Abcb1* genes, which are located in close proximity and are functionally related, a change in acetylated histone levels results in strikingly different changes in mRNA levels.

In summary, the observations we report here, together with several other reports on the regulation of ABC drug transporter expression, indicate that drug-resistant cells employ several mechanisms to increase MDR protein expression. Changes of histone modifications accompany altered *MDR* gene expression in drug-resistant cells, but careful analysis is warranted in order to determine whether those changes are in a cause-and-effect relationship with developing the multidrug resistance phenotype.
